# Extensive phenotypic characterisation of a human TDP-43^Q331K^ transgenic mouse model of amyotrophic lateral sclerosis (ALS)

**DOI:** 10.1038/s41598-021-96122-z

**Published:** 2021-08-17

**Authors:** Jodie A. Watkins, James J. P. Alix, Pamela J. Shaw, Richard J. Mead

**Affiliations:** Sheffield Institute for Translational Neuroscience, Department of Neuroscience, School of Medicine, University of Sheffield, Sheffield, UK

**Keywords:** Neurological disorders, Cell biology, Drug discovery, Neurology, Neuroscience, Molecular neuroscience, Motor control

## Abstract

The majority of preclinical studies in ALS have relied on transgenic models with overexpression of mutant human superoxide dismutase 1 (SOD1), widely regarded to have failed in terms of translation of therapeutic effects. However, there are still no widely accepted models of other genetic subtypes of ALS. The majority of patients show ubiquitinated cytoplasmic inclusions of TAR DNA binding protein of 43 kilodaltons (TDP-43) in spinal motor neurons at the end stage of disease and a small proportion have mutations in *TARDBP*, the gene encoding TDP-43. TDP-43 transgenic mouse models have been produced, but have not been widely adopted. Here, we characterised one of these models available from the Jackson Laboratory in detail. Compared to TDP-43^WT^ mice, TDP-43^Q331K^ mice had 43% less hindlimb muscle mass at 6 months and a 73% reduction in hindlimb compound muscle action potential at 8 months of age. Rotarod and gait analysis indicated motor system decline with elevated weight gain. At the molecular level, the lack of TDP-43 cellular pathology was confirmed with a surprising increase in nuclear TDP-43 in motor neurons. Power analysis indicated group sizes of 12–14 mice are needed to detect 10–20% changes in measured parameters with a power of 80%, providing valid readouts for preclinical testing. Overall, this model may represent a useful component of multi-model pre-clinical therapeutic studies for ALS.

## Introduction

Amyotrophic lateral sclerosis (ALS) is a neurodegenerative disease, causing progressive muscle weakness, spasticity and eventual respiratory failure, usually resulting in death within 3–5 years of symptom onset^1^.

Transactive response (TAR)-DNA-binding protein 43 (TDP-43) is a DNA/RNA binding protein, that is involved in mRNA processing, transcription and translational regulation. TDP-43 has been implicated in both ALS and frontotemporal lobar degeneration (FTLD). An overlap of the two diagnoses is increasingly recognised, with approximately 15% of patients with FTLD developing ALS and this extends to shared pathological, genetic and mechanistic features^2^. The main pathological finding in ALS is the presence of ubiquitinated inclusions of TDP-43 in the cytoplasm of both neurons and glia of the brain and spinal cord. These inclusions are found in most sporadic cases, as well as non-SOD1 familial ALS^3, 4^, regardless of whether the patient has a mutation in the *TARDBP* gene, which encodes the TDP-43 protein. However, several mutations in *TARDBP* have been identified as causal mutations associated with ALS^5–7^. Thus, approximately 97% of ALS cases show TDP-43 pathology, the only exception being familial cases with *SOD1* mutations^3^. This points to a possible mechanistic difference between SOD1-related ALS and all other subtypes of ALS. For this reason, the field urgently needs to identify a TDP-43 driven model for mechanism and therapeutic based studies. Such a model would complement the frequently used SOD1-related models.

Multiple TDP-43 mouse models have been generated to examine the role of TDP-43 in ALS^8^ Several overexpression models of wild-type or mutant TDP-43 have been investigated, resulting in a range of phenotypes including bodyweight changes, cytoplasmic TDP-43 inclusions, tremor, muscle atrophy, spasticity, impaired learning, impaired memory and premature death^9–14^. TDP-43 loss-of-function has also been modelled by conditional deletion of TDP-43 expression in adult mouse motor neurons, resulting in weight loss, muscle atrophy, motor neuron loss, cytoplasmic ubiquitinated TDP-43 inclusions and premature death^15, 16^. Thus, both increased and decreased expression of TDP-43 can lead to neuronal degeneration.

More recently a knock-in mouse model bearing a Q331K mutation in the endogenous mouse gene has been described, and is the most physiological model described to date, showing features of both ALS and FTD, with weight gain, lack of classical TDP-43 pathology and elevated nuclear TDP-43^17^.

Despite this extensive literature, no consensus has emerged as to which is the most suitable model, particularly for use in testing potential new therapeutic agents. There appear to be several reasons for this: the first is that transgenic overexpression of either mutant or wild-type TDP-43 causes neurodegeneration^13, 14^. Secondly, the most characterised model^18^ has a significant non-motor phenotype related to degeneration of autonomic neurons of the myenteric plexus, which causes premature death due to inhibition of gut transit^19–21^. Finally, the majority of models show considerable variability in the behavioural readouts, complicating their use in hypothesis driven or therapeutic research.

There is a need to characterise and validate TDP-43 driven models extensively, in order to determine their potential utility. Here, we investigate in detail the phenotypic characteristics of a mouse model transgenic for human *TARDBP* with a Q331K mutation (referred to as TDP-43^Q331K^), in comparison to wild-type TDP-43 (TDP-43^WT^), expressed under the murine prion promoter. This mouse model was originally described on a C57BL/6 J background^22^ but was deposited at the Jackson laboratories and subsequently backcrossed on to a different genetic background (C57BL/6 N).

In this study, we have undertaken a detailed assessment of this model. Our objectives were to confirm the previous findings on the new background, provide multiple quantitative readouts of disease progression, clearly define the motor phenotype and to outline the monitoring and assessment criteria for therapeutic trials and mechanistic assessments in this model.

## Results

### Abnormal gait evident at 27 weeks of age in TDP-43^Q331K^ mice

In both male and female mice, an intermittent resting tremor was evident by 10 weeks of age, which progressed to a pronounced continuous resting tremor by 14 weeks of age. Supplementary Movie 1 can be found online (supplementary data) and shows the resting tremor in a 6 month old TDP-43^Q331K^ female mouse.

**Figure 1:**
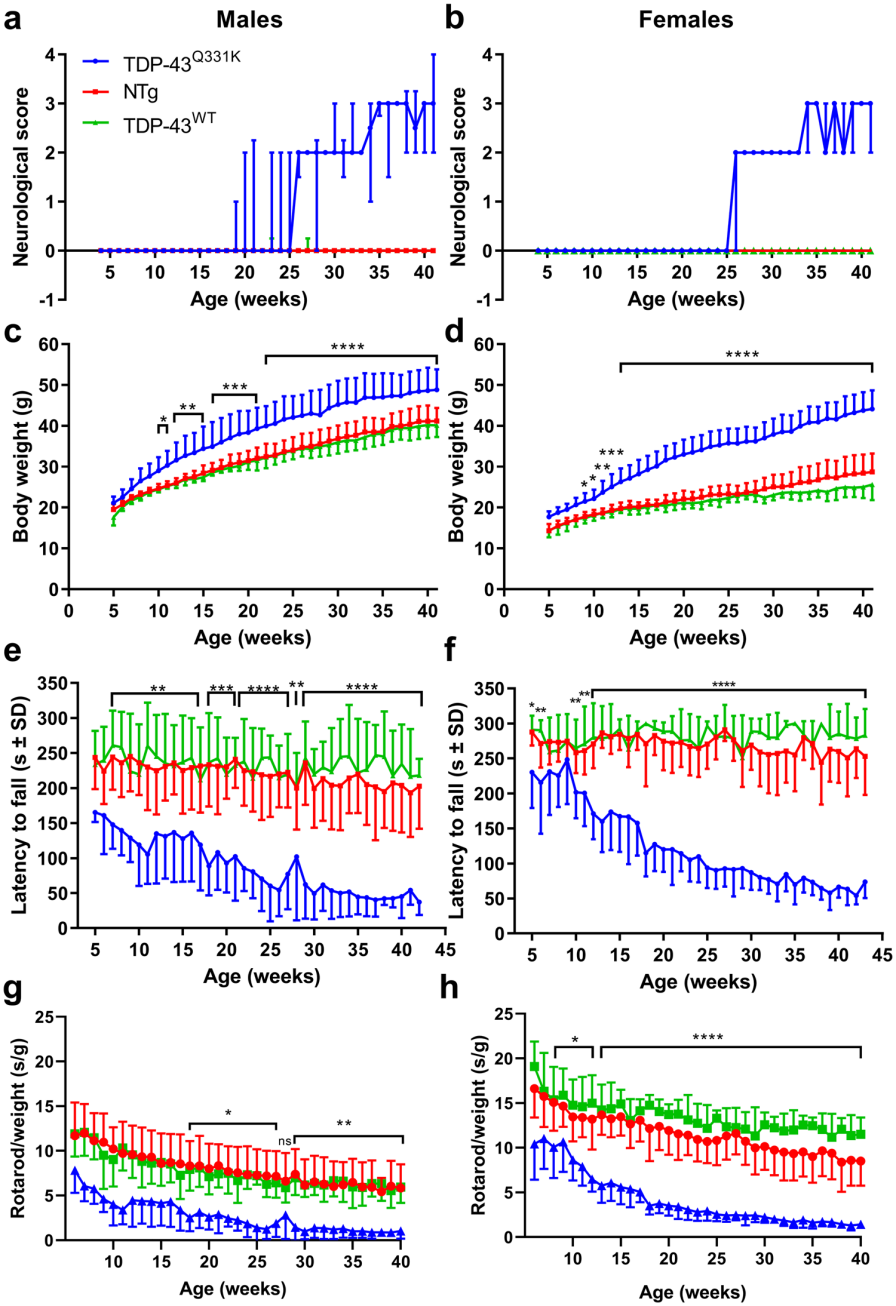
Body weight, CatWalk gait analysis and rotarod performance. (**A**) Male neuroscores. (**B**) Female neuroscores. (**C**) Male body weight. (**D**) Female body weight. (**E**) Male rotarod performance. (**F**) Female rotarod performance. (**G**) Male rotarod performance/weight. (**H**) Female rotarod performance/weight. Error bars represent mean ± SD. All graphs contain data for TDP-43^Q331K^ (n = 6 per gender—depicted by the blue lines), TDP-43^WT^ (n = 6 per gender—depicted by the green lines) and non-transgenic (n = 12 per gender—depicted by the red lines) mice compared using a two-way ANOVA with Tukey’s post-tests. *p < 0.05, **p < 0.01, ***p < 0.001, ****p < 0.0001 TDP-43^Q331K^ vs all other groups.

Male and female TDP-43^Q331K^ mice developed an abnormal gait (neuroscore of 2) at around 27 weeks of age and progressed to develop an obvious waddling or “swimming” gait (Fig. 1A,B, Supplementary Movie 2, online) which can be quantified using gait analysis (see below). Conversely, TDP-43^WT^ (Supplementary Movie 3, online) and non-transgenic mice showed no sign of tremor or gait abnormality (non-transgenic not shown).

### TDP-43^Q331K^ show weight gain compared to TDP-43^WT^ mice

TDP-43^Q331K^ mice were significantly heavier than non-transgenic and TDP-43^WT^ from 10 weeks of age in males (TDP-43^Q331K^ 29 ± 3.3 g vs TDP-43^WT^ 24.4 ± 0.7 g vs non-transgenic 24.7 ± 1.1 g, *p* < 0.05 overall, by two-way ANOVA, see figure for post-tests) and 9 weeks of age in females (TDP-43^Q331K^ 21 5 ± 2.0 g vs TDP-43^WT^ 17.4 ± 1.3 g vs non-transgenic 17.8 ± 1.1 g, *p* < 0.05, overall, by two-way ANOVA, see figure for post-tests). This phenotype was not described originally in this model. The difference in weight continued to increase with age (Fig. 1C,D).

### Rotarod performance decreases with age in the TDP-43^Q331K^ mice

TDP-43^Q331K^ mice showed an age-dependent decrease in latency to fall on the accelerating rotarod compared to non-transgenic and TDP-43^WT^ mice by 7 weeks of age in males (TDP-43^Q331K^ 147.8 ± 34.6 s vs TDP-43^WT^ 261.0 ± 49.4 s vs non-transgenic 244.3 ± 46.7, *p* < 0.01, two-way ANOVA) and 10 weeks of age in females (TDP-43^Q331K^ 201.6 ± 36.9 s vs TDP-43^WT^ 264.8 ± 40.8 s vs non-transgenic 258.3 ± 29.2 s, *p* < 0.01, two-way ANOVA) (Fig. 1E,F).

It is possible that the rotarod findings were influenced by the weight of the mice, as a previous study reported a correlation between increased weight and decreased rotarod performance^23^. Therefore, we corrected for weight by plotting the ratio of latency to fall (seconds)/weight (g) and the significant difference in rotarod performance found in the TDP-43^Q331K^ mice was still evident (Fig. 1G,H).

### Gait analysis

**Figure 2:**
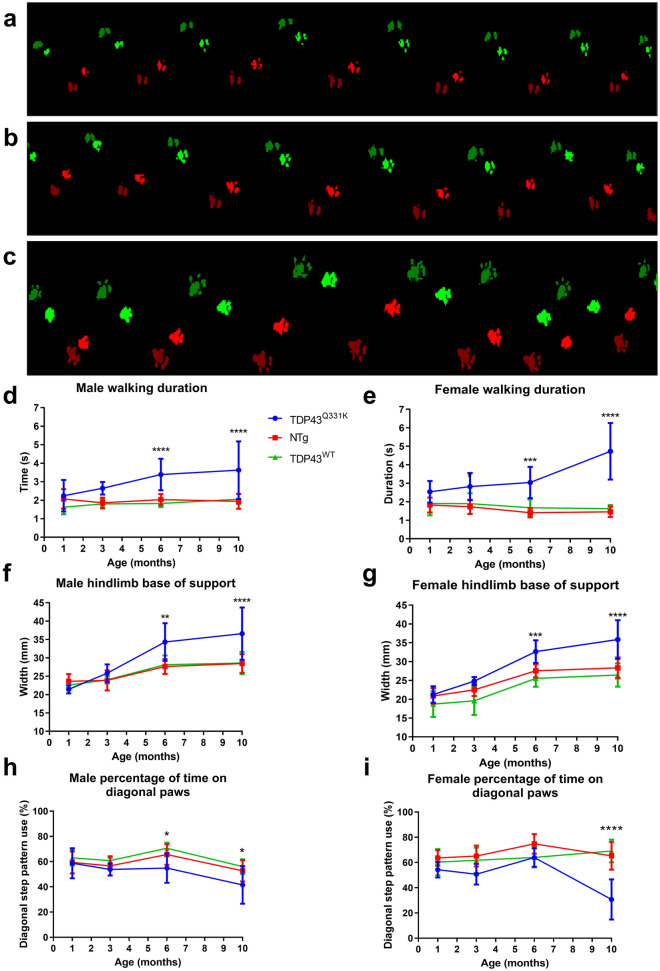
CatWalk gait analysis showing paw print patterns; walking duration, hindlimb base of support and diagonal pattern walking in TDP-43^Q331K^ mice compared to TDP-43^WT^ and non-transgenic mice. Footprint traces of non-transgenic (**A**), TDP-43^WT^ (**B**) and TDP-43^Q331K^ (**C**) mice at 10 months of age. Red prints = right limbs, green prints = left limbs, bright prints = forelimbs, darker prints = hindlimbs. In TDP-43^Q331K^ n = 6 males, 6 females, NTg n = 12 males, 12 females, TDP-43^WT^ n = 6 males, 6 females. Analysis of individual gait parameters over time in the TDP-43^Q331K^, TDP-43^WT^ and non-transgenic mice. Mean (± SD) walking duration (time to walk a distance of 43.8 cm) in seconds was measured in males (**D**) and females (**E**). Mean (± SD) hindlimb base of support (distance between hind paws) was measured in males (**F**) and females (**G**). Mean (± SD) diagonal stepping pattern in males (**H**) and females (**I**). *p < 0.05 vs all other groups, **p < 0.01 vs all other groups, ***p < 0.001 vs all other groups, ****p < 0.0001 vs all other groups, two way ANOVA with Tukey's post-tests*.*

Using the CatWalk gait analysis system, it was possible to identify a number of gait abnormalities. Paw prints were wider, with an unsteady gait pattern in the TDP-43^Q331K^ mice (Fig. 2C), compared to the gait of non-transgenic (Fig. 2A) and TDP-43^WT^ transgenic mice (Fig. 2B). Walking duration (Fig. 2D,E) increased significantly in TDP-43^Q331K^ mice by 6 months of age (*p* < 0.001) and this slowing of gait is more prominent by 10 months of age (*p* < 0.0001).

The hind-limb base of support is the distance between left and right hindlimbs perpendicular to the direction of travel and captures the severe waddling or swimming gait of the TDP-43^Q331K^ mice. This measurement increases from ~ 21 mm to ~ 36 mm between 1 and 10 months of age in both male and female TDP-43^Q331K^ mice and this increase is statistically greater than that seen in both sets of control mice at 6 and 10 months of age (Fig. 2F,G).

A useful indicator of unsteady gait is the percentage of time spent on diagonal paws, the predominant stepping pattern in healthy mice. The percentage of time spent on diagonal paws is decreased in male mice at 6 and 10 months of age (*p* < 0.05) and at 10 months of age in female mice indicating an increasingly unsteady gait with age (*p* < 0.0001) (Fig. 2H,I).

### Lumbar spinal motor neurons show increased levels of c-myc-tagged TDP-43

**Figure 3:**
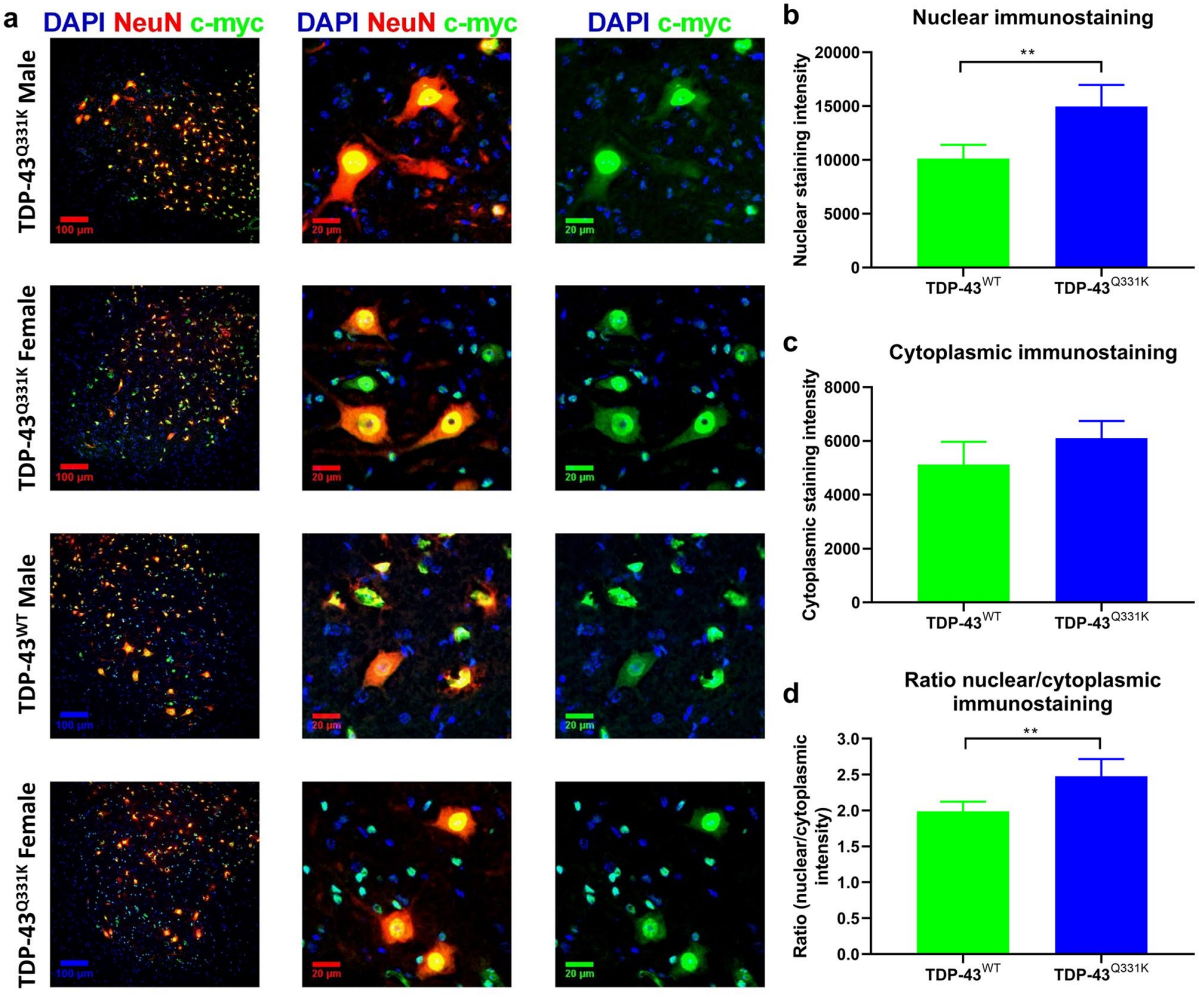
Immunohistochemical staining of lumbar spinal cord to detect human TDP-43. Immunohistochemical staining of DAPI, NeuN and c-myc in the lumbar ventral horns of 10 month old TDP-43^WT^ and TDP-43^Q331K^ male and female mice (n = 5/group). Panels in (**A**) show example staining from one male TDP-43^Q331K^, one male TDP-43^WT^, one female TDP-43^Q331K^ and one female TDP-43^WT^ lumbar spinal cord. Scale bars are 100 µm (first column), and 20 µm (columns 2 and 3). Nuclear (**B**) and cytoplasmic (**C**) quantification using automated pixel intensity counting (± SD) of c-myc-tagged hTDP-43 in the lumbar cord. (**D**) Ratio of nuclear/cytoplasmic staining of c-myc-tagged hTDP-43. Error bars represent mean ± SD, one way ANOVA, ** = *p* < 0.01.

Lumbar spinal cord sections from TDP-43^WT^ and TDP-43^Q331K^ mice showed clear nuclear staining (Fig. 3A) for c-myc-tagged human TDP-43 (green). C-myc-tagged TDP-43 was also seen in the cytoplasm of motor neurons, identified by their size, morphology and location. Nuclear staining intensity of c-myc-tagged TDP-43 was significantly higher in TDP-43^Q331K^ spinal cord motor neurons (*p* < 0.01) (Fig. 3B). However, there was no significant difference in cytoplasmic staining intensity of c-myc-tagged TDP-43 between groups (Fig. 3C). Surprisingly, the ratio of nuclear/cytoplasmic staining intensity of c-myc-tagged TDP-43 was also significantly higher in TDP-43^Q331K^ motor neurons (*p* < 0.01) (Fig. 3D).

### Human TARDBP transgene and TDP-43 protein expression

**Figure 4:**
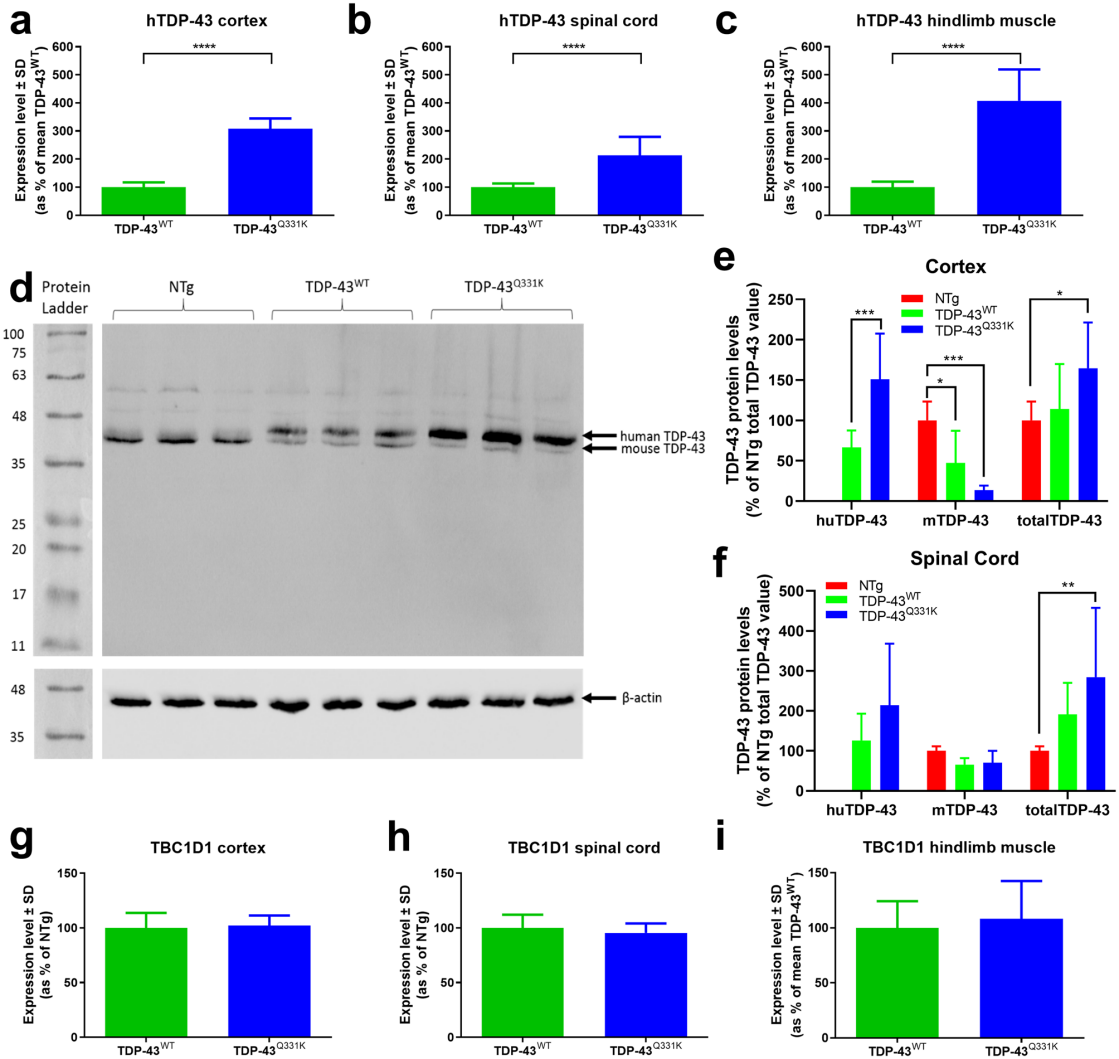
hTDP-43 expression, hTDP-43 and mTDP-43 protein quantification, Tbc1d1 expression. (**A**–**C**) RT-qPCR for hTARDBP expression in TDP-43^Q331K^ mice as a percentage of the mean TDP-43^WT^ in cortex (**A**), spinal cord (**B**) and hindlimb muscles (**C**). ****p < 0.0001, Student’s t test. (**D**) Western blot showed two separate bands for human (44.2kD) and mouse TDP-43 (43kD). This example shows non-transgenic, TDP-43^WT^ and TDP-43^Q331K^ 10 month old cortex samples respectively (n = 3, 1 animal per lane). Note the same blot was processed and captured separately for TDP-43 and β-actin staining and these images are separated by white lines. Likewise the protein ladder lane was captured separately under normal illumination and aligned with the blot image. (E–F) Relative human, mouse and total TDP-43 protein levels in 10 m non-transgenic (n = 10 males, 9 females), TDP-43^WT^ (n = 5 males, 5 females) and TDP-43^Q331K^ (n = 4 males, 5 females) mice in cortex (**E**), and spinal cord (**F**). All samples are normalised to the non-transgenic total TDP-43 value for each Western blot. (**G**–**I**) Tbc1d1 expression in TDP-43^Q331K^ mice (n = 4 males, 5 females) as a percentage of the mean TDP-43^WT^ levels (n = 5 males, 5 females) in cortex (**G**), spinal cord (**H**) and hindlimb muscle (**I**) showed no differences on a student’s t test. All data shown are mean ± SD.

The original report describing this model indicated that the level of expression of the introduced transgene and protein were not significantly different between the TDP-43^Q331K^ and TDP-43^WT^ lines^22^. In order to compare the level of transgene expression between lines, human TDP-43 (huTDP-43) transcript levels were measured by RT-qPCR in the cortex, spinal cord and hindlimb muscles of TDP-43^Q331K^ and TDP-43^WT^ mice at 10 months of age (Fig. 4A–C). Expression of huTDP-43 transcripts was over threefold higher in the TDP-43^Q331K^ cortex compared to the TDP-43^WT^ cortex (308.4 ± 36.4% vs 100 ± 17.3%, *p* < 0.0001), twofold higher in the TDP-43^Q331K^ spinal cord compared to the TDP-43^WT^ spinal cord (213.3 ± 65.8% vs 100 ± 13.3%, *p* < 0.0001), and fourfold higher in the TDP-43^Q331K^ hindlimb muscle compared to the TDP-43^WT^ hindlimb muscle (407.6 ± 111.9% vs 100 ± 19.8%, *p* < 0.0001).

Western blotting with an anti-TDP-43 antibody allowed for quantification of both human and mouse TDP-43 protein due to the c-myc tag on the human protein increasing its molecular weight (Fig. 4D–F). Moderate levels of human TDP-43 were measured in the TDP-43^WT^ mice at 10 months of age (66.6 ± 21.0% in cortex, 126.1 ± 67.1% in spinal cord compared to endogenous mouse TDP-43), whilst levels in the TDP-43^Q331K^ mice were significantly higher than endogenous mouse TDP-43 in cortex (151 ± 56.5%, *p* < 0.001) but not in spinal cord (213.9 ± 154.1%). Transgenic expression of human TDP-43 resulted in a significant reduction in endogenous mouse TDP-43 in the TDP-43^WT^ cortex (47.5 ± 39.6% in cortex, *p* < 0.05) but not significantly in the spinal cord (65.5 ± 16.3%) compared to non-transgenic. Expression of endogenous TDP-43 was significantly reduced in the cortex of TDP-43^Q331K^ mice (13.6 ± 5.6%, *p* < 0.001) but not in spinal cord (70.7 ± 29.6%). Total TDP-43 levels (mouse plus human) were significantly higher in the TDP-43^Q331K^ mice (164.6 ± 56.6% in cortex, *p* < 0.05, 284.5 ± 173.3% in spinal cord, *p* < 0.01, versus endogenous mouse), compared to non-transgenic mice but not significantly higher than in the TDP-43^WT^ mice (114.1 ± 55.8% in cortex, 191.6 ± 78.5 in cord).

### *Tbc1d1* gene expression was equal in all tissues measured

In previous studies conditional knockout of *tardbp* caused leanness and lethality due to altered fat metabolism as a result of reduction in *tbc1d1* levels, a known regulator of glucose 4 transporter (GLUT4) translocation in skeletal muscle^24^. Transgenic overexpression of TDP-43 with an A315T mutation had the opposite effect with increased fat deposition, which correlated with elevated *tbc1d1* expression^25^. In this study, *tbc1d1* gene expression was not altered between mice expressing mutant or wild-type TDP-43 in cortex, spinal cord or muscle between groups (Fig. 4G–I).

### TDP-43^Q331K^ mice lose over 40% of lower hind limb muscle mass

**Figure 5:**
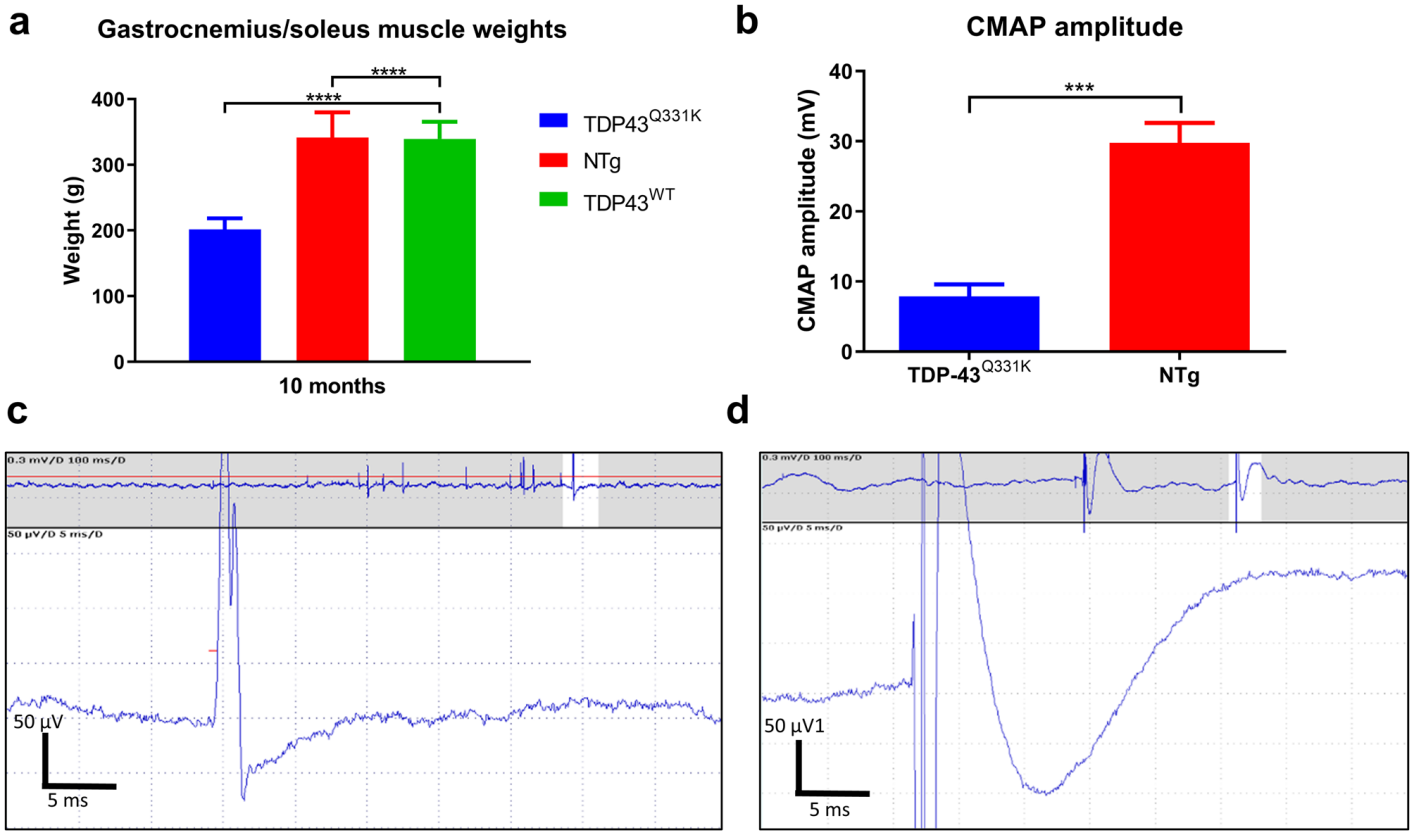
Muscle weights and electrophysiology. (**A**) Mean (± SD) gastrocnemius/soleus muscle weights of female mice at 10 months of age TDP-43^Q331K^ n = 10, NTg n = 12, TDP-43^WT^ n = 6. (**B**) Compound muscle action potential (CMAP) of the lower hindlimb of TDP-43^Q331K^ mice and non-transgenic mice (± SD) at 9–11 months of age (n = 3). ***p < 0.001 vs all other groups, ****p < 0.0001 vs other groups, one way ANOVA with Tukey's post-tests. (**C**, **D**) Fasciculation potentials recorded in two out of eight TDP-43^Q331K^ mice tested. Such electrophysiological activity was not seen in TDP-43^WT^ mice. Mice were tested for CMAP and fasciculation at the same time point.

A significantly lower hindlimb muscle weight was evident in TDP-43^Q331K^ mice compared to the TDP-43^WT^ and non-transgenic mice at 10 months of age (201.4 ± 17.2 mg TDP-43^Q331K^ vs 339.7 ± 25.8 mg TDP-43^WT^ vs 341.5 ± 38.3 mg, *p* < 0.0001, one-way ANOVA), (Fig. 5A).

### TDP-43^Q331K^ mice have reduced compound muscle action potential (CMAP) amplitude and fasciculations

In order to confirm that the loss of muscle mass correlated with reduced electrophysiological properties, CMAP amplitude was measured. A significant reduction in CMAP amplitude in the TDP-43^Q331K^ mice compared to non-transgenic mice was evident in mice aged 9–11 months (7.9 ± 1.7 mV vs 29.8 ± 2.8 mV, *p* < 0.001, two-way ANOVA). This represents a 73% reduction in CMAP amplitude in the TDP-43^Q331K^ mice (Fig. 5B). Loss of CMAP amplitude can be a non-specific observation relating, for example, to motor axon loss, or muscle dysfunction. To explore the aetiology of CMAP data we undertook hindlimb electromyography (EMG) in 8 mice. Clear fasciculation potentials were recorded in 2 of the eight mice (Fig. 5C,D).

### Optimal study design based on statistical power analysis

One of the key determinants of model utility is the ability to detect therapeutic or other intervention effects with relatively high statistical confidence with reasonable group sizes. For the data generated here we undertook statistical power analysis to identify numbers of animals needed to detect a range of effect sizes (Table 1). We focussed on female mice overall as they showed less variability in the various readouts (e.g. see Fig. 1) meaning that smaller group sizes would be needed for a given effect size and statistical power. We calculated coefficients of variation (CV) for each readout and these are low overall with none above 16% and 6/8 below 11% (Table 1). Power analysis assuming a two-tailed Student’s *t* test with α = 0.05 and β 0.8 was performed on 6 and 10 month data with effect sizes between 10 and 20%. We have previously used time taken to reach a 20% decline in performance for rotarod data,^26^ and so we used this parameter in the present study as it mitigates the relatively high variability seen on individual timepoints. We noted that a 10 month study does not provide significant additional information over a study length of 6 months, with the exception that time to reach a neuroscore of 2 would be missed. A group size of 14 per group is sufficient to capture the lowest effect size (10%) for all measured parameters. Thus, overall we would recommend group sizes of 14 with a 6 month study duration as being able to capture low effect sizes with reasonable confidence in as short a study as possible.

**Table 1:** Numbers of mice/group required for investigation of each parameter.

Parameter	Age (months)	Mean values in TDP-43^Q331K^ mice (± SD)	Coefficient of variation (%)	Mean difference TDP-43^WT^ vs TDDP-43^Q331K^ (%)	N required to detect an x% improvement
Time to reach a neuroscore of 2 (severe waddle)	N/A	26.4 weeks ± 0.5	2.0	N/A	10% = 3
					15% = 2
					20% = 2
Weight	6	35.3 g ± 3.7	10.4	+ 55	10% = 14
					15% = 7
					20% = 5
	10	43.8 g ± 4.4	10.1	+ 54	10% = 14
					15% = 7
					20% = 5
Increase by 2 weeks in time to reach a 20% decline in rotarod performance from maximum	N/A	11.4 weeks ± 1.8	15.9	N/A	11
CatWalk hindlimb base of support	6	32.7 ± 3.0	9.2	+ 28	10% = 12
					15% = 6
					20% = 4
	10	35.8 ± 5.1	14.3	+ 36	10% = 27
					15% = 13
					20% = 8
Gastrocnemius/ soleus muscle weight	6	175.5 ± 7.8	4.4	− 43	10% = 4
					15% = 3
					20% = 2
	10	201.4 ± 17.2	8.5	− 41	10% = 10
					15% = 5
					20% = 4

All power analyses are based on detecting a power of 80% in females, using the highest standard deviation (SD) from the relevant time-point.

## Discussion

For many years, models based on overexpression of mutant SOD1^27^ have been the major workhorse for preclinical testing in ALS. This model has provided useful insights, however, the lack of translation of therapeutic effects from this model to clinical ALS indicates a need for new therapeutic testing paradigms^28^. This may be particularly true given that SOD1-related ALS is not a TDP-43 proteinopathy^3^. Previously, serious flaws in experimental design were identified with the majority of studies in SOD1 related models^29^, mandating that new models must be carefully characterised and serious attention paid to study design. There are currently nine TDP-43 mouse models readily available at The Jackson Laboratory (USA) for which the original papers are available, including the model described here.

Two models overexpressing wild type TDP-43^12, 13^ showed a dose-dependent phenotype with some evidence of TDP-43 pathology, indicating that overexpression of TDP-43 alone can cause an ALS-like disease. Mutant TDP-43 models which highly overexpress the *TARDBP* gene show severe phenotypes^9, 30^. It has been reported that a truncated TDP-43 model has a mild motor phenotype, but the TDP-43 protein was unaffected and the truncation is not one found in ALS patients^31^. Aggressive disease progression and early death has been an issue for several models including two with an M337V mutation under the murine prion promoter^14, 30^ and a postnatal deletion of TDP-43 which caused a fatal increase in fat metabolism^24^. A mouse model with an A315T mutation expressed using the murine prion promoter^18^ was the first published and has been studied by multiple groups. However this mouse suffers from gut immotility, due to increased huTDP-43 expression in the myenteric plexus and degeneration of neurons therein, complicating the phenotype^19–21, 32^.

The TDP-43 mouse model described in this paper has a minimal overexpression of the human *TARDBP* gene for both the mutant and wild-type transgenics, where the authors targeted a level of expression similar to endogenous mouse TDP-43^22^. Furthermore, the Q331K mutation has been found in patients^7^, and these mice showed no signs of gastrointestinal problems.

In contrast to the original study describing these mice we found that the level of expression of transcripts encoding human TDP-43 were higher in TDP-43^Q331K^ transgenic mice compared to TDP-43^WT^ mice in both cortex and spinal cord and higher at the protein level in cortex. This calls into question whether this is a mutant specific phenotype, one of the initial attractions in characterising this model and should be considered when selecting controls.

Despite this, the TDP-43^Q331K^ mouse on the C57BL/6 N background shows promise as a model of ALS as it demonstrates signs of reduced muscle mass, muscle weakness and motor dysfunction. The most interesting aspect of these findings is that they occur without the presence of canonical TDP-43 proteinopathy. It is conceivable that the model will develop TDP-43 pathology at a later time-point, as shown in a similar strain of TDP-43^Q331K^ on a C57BL/6 J background at 24 months of age^33^. These mice (line 31) were generated at the same time as the line tested here (line 103) and are described briefly in the Arnold et al. paper^22^ although not characterised. Therefore, it is possible that the findings we present here are representative of an early stage of disease, in which a motor phenotype is evident prior to mislocalisation and aggregation of TDP-43. The development of an early phenotype is advantageous as it will allow us to investigate the onset and early stages of disease, as well as providing the potential to investigate disease modifiers such as gene-environment and gene–gene interactions.

Previous investigations of TDP-43^Q331K^ mice on a C57BL/6 J background found no evidence of nuclear depletion of TDP-43^22^. We found increased cytoplasmic TDP-43 in the TDP-43^Q331K^ mice compared to the TDP-43^WT^ mice, at 10 months of age, as described previously in a similar model at 24 months of age^33^. However, we also report increased nuclear TDP-43 in the TDP-43^Q331K^ mice compared to the TDP-43^WT^ mice. Overall this may suggest that mislocalisation per se is not required for the onset of motor system degeneration, but the absolute level of TDP-43 in any one compartment (cytoplasmic or nuclear) may be the driving factor. This would fit in with a dominant negative model of TDP-43 dysfunction^34^. This finding has been highlighted also in a knock-in mouse model where the Q331K mutation has been introduced into the endogenous mouse *tardbp* gene^17^. Interestingly these mice also demonstrate increased weight gain and a lack of TDP-43 mislocalisation. This may suggest that mild overexpression of TDP-43 via a transgenic approach is physiologically relevant and we believe these findings have wider implications for TDP-43 biology.

The results we present here consistently demonstrate a detectable decline in motor performance by 6 months of age in the TDP-43^Q331K^ mice. Progressive gait abnormalities become identifiable on neuroscoring at around 6 months of age, consistent with the significant changes seen in hindlimb base of support and walking duration on quantitative gait analysis, and a 40% loss of gastrocnemius muscle mass.

Rotarod performance is significantly reduced in the TDP-43^Q331K^ mice by 10 weeks of age, consistent with findings in a previous study of TDP-43^Q331K^ mice on a C57BL/6 J background, in which the mice were found to have a significantly reduced rotarod performance at 3 months of age^22^.

We observed that ‘walking duration’, increases as TDP-43^Q331K^ mice age when compared to both TDP-43^WT^ and non-transgenic mice. This suggests a reduction in speed which may be related to motor dysfunction and/or a reduced motivation. In addition, during normal gait, mice predominantly walk with diagonally opposite paws in contact with the ground at any one time in a diagonal stepping pattern. Both male and female TDP-43^Q331K^ mice showed a reduction in this gait type with aging suggesting increasingly unsteady gait with age.

A link between manipulation of TDP-43 and *tbc1d1* expression has previously been described in mouse models where deletion of *tardbp* decreased *tbc1d1* expression and transgene mediated overexpression of *tardbp* lead to increased *tbc1d1* levels^24, 25^. The *tbc1d1 gene* encodes a regulator of the translocation of Glut4, a glucose transporter and so this has been proposed as the mechanism by which TDP-43 regulates fat deposition^24, 25^.

We observed increased weight gain in the TDP-43^Q331K^ model described here with mild overexpression of mutant TDP-43 and investigated whether *tbc1d1* gene expression was also elevated as a potential mechanism causing weight gain. Levels of *tbc1d1* expression were similar between TDP-43^Q331K^ mice when compared to TDP-43^WT^ mice in cortex, spinal cord and hindlimb muscles. In this model at least there is no correlation between levels of huTDP-43, *tbc1d1*, and the observed weight gain. Further work is needed to identify the mechanism of weight gain. However, we know that TDP-43 pathology defines subtypes of FTD and ALS-FTD patients, in contrast to ALS patients, show significantly elevated BMI^35, 36^ and a recent TDP-43^Q331K^ non-overexpressing model also showed weight gain, which was hypothesised as an FTD-related feature as suggested by other FTD-like phenotypes in this model^17^.

CMAP amplitude quantifies the depolarisation of muscle fibres and a reduction in CMAP amplitude may correlate with loss of functional motor axons, as is often seen in ALS patients^37^. CMAP amplitude was significantly lower in TDP-43^Q331K^ mice compared to non-transgenic mice at 9–11 months of age. This may indicate either fewer functional motor axons, as has been demonstrated previously in the SOD1^G93A^ mouse model^38, 39^, or functional deficits at the level of the neuromuscular junction or muscle. EMG recordings at 6 months revealed fasciculation potentials, evidence of neurogenic (as opposed to myopathic) dysfunction.

A recent study used the same TDP-43^Q331K^ mice described here to investigate the effect of heterozygous deletion of *Sarm 1* on axonal degeneration. Sarm1 is activated during Wallerian degeneration and its deletion was shown to protect motor axons and neuromuscular junctions from degeneration. The mice were also shown to display FTD-like phenotype with enhanced weight gain, brain atrophy and cognitive impairments^40^.

Our findings show consistent disease progression in both male and female TDP-43^Q331K^ mice. The data from the female mice are less variable, particularly for weight and rotarod performance. This indicates that, in some studies, it may be more efficient to use females alone.

Statistical power analysis for estimation of group sizes needed to detect different effect sizes indicated that differences of 10% can be detected at 6 months of age in neuroscoring, body weight, CatWalk hindlimb base of support and gastrocnemius/soleus muscle weights with group sizes of 14 for a pair-wise analysis (Student’s T Test). An extension of 2 weeks in time to reach a 20% decline in rotarod performance would require 11 mice per group. Therefore, we recommend group sizes of 14 females to detect any potential disease-modifying effect in a 6 month therapeutic study with reasonable power (80%). The reliability of multiple readouts of motor system function makes the model attractive for researchers interested in pursuing therapeutic or mechanistic studies. The ‘3Rs’ of reduction, replacement and refinement are critical in preclinical research, and we demonstrate that a large body of data can be obtained with 14 animals per group, which compares favourably with the group sizes recommended in the SJLBL6 SOD1^G93A^ mice of 24 per group^29^. Furthermore, refinement is applied by using a mild disease model in which mice do not reach a distressing ‘end-stage’ and we suggest that therapeutic studies do not need to continue beyond 6 months.

In summary, this study provides a comprehensive analysis of this TDP-43^Q331K^ transgenic model which should enable other laboratories to include it as a valid system for preclinical screening cascades with a view to clinical translation. The model has a number of advantages over other TDP-43 based systems. Firstly, it is a robust and reproducible model without confounding effects on gut motility. A range of quantitative readouts show low variability enabling well powered studies with reasonable group sizes for statistical analysis. It recapitulates features (increased nuclear TDP-43, weight gain) described in a model with an endogenous TDP-43^Q331K^ mutation^17^, confirming that it is physiologically relevant. Overall the wider adoption of this model should improve assessment of novel therapeutic strategies for ALS, a key goal of translational ALS research.

## Materials and methods

Please note that the material and methods section was adapted from the PhD Thesis of the first author^41^ which can be found at http://etheses.whiterose.ac.uk/id/eprint/16694 re-used here under a creative commons CC BY-NC-ND 2.0 UK license (https://creativecommons.org/licenses/by-nc-nd/2.0/uk/).

### Ethics statement

All mouse experiments were carried out under the terms of the UK Animals (Scientific Procedures) Act 1986 and under a UK Home Office project license and ethically reviewed and approved by the University of Sheffield Animal Welfare and Ethical Review Body (AWERB). Mice were housed and cared for in accordance with the Home Office Code of Practice for Housing and Care of Animals Used in Scientific Procedures. ARRIVE guidelines^42^ were adhered to in the preparation of this report^41^.

### Transgenic C57BL/6 N mice

TDP-43^WT^ (line 96) and TDP-43^Q331K^ mice (line 103) *(Mus musculus)* were originally generated on a C57BL/6 J background by insertion of c-myc-tagged human *TARDBP*^22^ and were subsequently backcrossed on to a C57BL/6 N background before being deposited at the Jackson Laboratory (stock numbers 017907 and 017933, respectively).

The basic characterisation tests were carried out with 5–7 males and 5–7 females per group (TDP-43^WT^ and TDP-43^Q331K^), non-transgenic littermates of each mouse were also tested, the data for which were pooled to create the non-transgenic (NTg) group. Mice were recruited at 25 days of age.

The electrophysiology pilot study consisted of 3 female TDP-43^Q331K^ mice and 3 female NTg mice at ages ranging from 9 to 11 months.

Tissue was collected for histological investigations at 1, 3, 6 and 10 months of age, collecting from 6 mice (3 male, 3 female) from each group (non-transgenic, TDP-43^WT^, TDP-43^Q331K^) for each time point^41^.

### Housing

Mice were bred in a specified pathogen free environment, and transferred to a conventional facility for the studies described here. The facility uses a 12 h light/dark cycle, and room temperature is set to 21 °C. Cages were lined with fine sawdust (Eco-Pure Flakes 6, Datesand, Manchester, UK), which was changed weekly, a plastic house was placed in each cage and paper wool (Datesand, Manchester, UK) was used as bedding material. Mice were fed 2018 rodent diet (Harlan, Bicester, UK) ad libitum. Water was provided ad libitum and changed weekly. Fresh bottles were provided every 2 weeks.

All mice were housed in same-sex littermate pairs or quadruplets. However, some male pairs were separated at around 6 months of age due to fighting, and were subsequently housed singly^41^.

### Genotyping

Mice were identified by ear clipping and the ear clip was then used for genotyping. DNA was extracted by incubating ear clips at 65 °C for 15 min in a 20 µl volume of QuickExtract DNA Extraction Solution (Epicentre, Cambridge, UK), followed by a 2 min incubation at 98 °C. 1 µl of DNA was then placed in solution with 4 µl of Firepol (Solis Biodyne, Tartu, Estonia), 11 µl of water, 0.5 µm of each huTDP-43 primer (forward 5′-AGAGGTGTCCGGCTGGTAG-3′, reverse 5′-CCTGCACCATAAGAACTTCTCC-3′) and 0.5 µm of each interleukin-4 receptor control primer (forward 5′-CTAGGCCACAGAATTGAAAGATCT-3′, reverse 5′-GTAGGTGGAAATTCTAGCATCATCC-3′).^41^. Transgene copy number was not assessed in the mice on this study.

### Neuroscoring and weighing

Neuroscores and weight were monitored weekly in the morning, measuring forelimb and hindlimb tremor on a scale of 0–3 (normal-strong). A ‘neuroscore’ scale was used to delineate motor dysfunction (0-normal, 1-significant hindlimb tremor, 2-abnormal gait, 3-severe waddle, 4-dragging of one hindlimb). These measurements were taken immediately prior to rotarod testing and in the same procedure room as the rotarod^41^.

### Accelerating Rotarod test

Mice were place on the accelerating rotarod (Jones & Roberts for mice 7650) for a period of up to 300 s. In this time the rotarod accelerates from 4 to 40 rpm and latency to fall was recorded for each mouse. All rotarod testing was carried out at a similar time of day (am) and all testing consisted of two trials, with the highest score being recorded for analysis.

Mice were initially trained on the rotarod over 3 days (27–29 days of age) and underwent the first testing day at 30 days of age, being tested each week thereafter^41^.

### Gait analysis

The CatWalk gait analysis system 7.1 (Noldus Information Technology B.V., Wageningen, Netherlands) was used to capture gait parameters at 1, 3, 6 and 10 months of age. Mice were placed on the glass floor of the CatWalk system in complete darkness and left to walk/run freely. Whenever possible, 6 runs were recorded and 3 runs were selected for analysis. CatWalk software 7.1 was used to analyse the gait parameters of the mice. Walking duration is the time taken to cross the length of the recording window (43.8 cm)^41^.

### Tissue collection for immunohistochemical staining

Mice were sacrificed by trans-cardiac perfusion following anaesthetic overdose using an intraperitoneal injection of pentobarbitone (2.5 ml/kg). Under deep anaesthesia (checked by absence of pedal reflex) and prior to cessation of cardiac function, a thoracotomy was performed, in which the left ventricle was cannulated with a syringe containing PBS and mild pressure applied to distend the right auricle, which was then cut and the mouse was perfused with phosphate buffered saline (PBS) via the left ventricle of the heart, followed by perfusion with 4% PFA/PBS.

Spinal columns were then extracted and placed in 4% PFA/PBS overnight. Next the spinal cord was extracted from the spinal column and cryoprotected by immersion through a gradient of 5, 10, 15 and 20% sucrose/PBS for 5 min in each concentration before overnight incubation in 20% sucrose overnight. The spinal cords were then dissected into cervical, thoracic, lumbar and sacral segments, and frozen in OCT embedding matrix (CellPath, UK) over dry ice, then stored at − 80 °C .

14 μm lumbar sections were sliced using a cryostat and mounted on to uncoated, charged slides (CellPath, Newtown, UK). Sections were mounted serially over a series of 5 slides, with 5 sections per slide, then stored at − 20 °C^41^.

### Immunohistochemical staining for c-myc and NeuN

Slides were placed at 37 °C for 30 min and then washed three times in PBS at room temperature with shaking. Sections were then permeabilised in 5% BSA, 0.5% triton X-100/PBS for 90 min at 37 °C and subsequently placed in an antigen access unit (A. Menarini Diagnostics, Winnersh, UK) submerged in Access Revelation (MP-607- × 500, A. Menarini Diagnostics, Winnersh, UK) for a 30 min cycle at 125 °C, with a pressure of 20 psi.

After washes in PBS, slides were blocked with 5% BSA, 0.25% Triton X-100/PBS for 10 min and then incubated with the primary antibodies diluted in 0.25% Triton X-100/PBS overnight at 4 °C (polyclonal rabbit anti-c-myc [Sigma catalogue #C3956, batch #093M4774V], monoclonal mouse anti-NeuN [Millipore catalogue #MAB377, batch #2592741] at 1:1000).

Following washes in PBS, slides were blocked in 5% BSA/PBS for 10 min and then incubated with secondary antibodies at room temperature for 90 min (Alexa Fluor donkey anti-rabbit 488 [Life Technologies A21206], Alexa Fluor donkey anti-mouse 555 [Life Technologies A31570], both at 1:1000 in 1% BSA).

Slides were then washed in PBS and filtered water. Sections were mounted in Vectashield hard set aqueous mountant with 4',6-diamidino-2-phenylindole (DAPI) (Vector Laboratories, Peterborough, UK) and left to dry at 4 °C overnight.

Images were captured using the INCell Analyzer 2000 (GE Healthcare, Hatfield, UK) and analysed using INCell analysis software. Image collection and analysis was carried out blinded. Images were produced using ImageJ^41^.

### Tissue collection

Mice were sacrificed at 10 months of age by overdose using an intraperitoneal injection of pentobarbitone (2.5 ml/kg) and exsanguination via cardiac puncture using a 25 gauge 0.5 × 16 mm needle (BD microlance, Ireland) and a 1 ml syringe (Terumo Europe, Belgium), after cessation of the pedal reflex. The brain was removed and the blunt edge of a steel back industrial single edge blade (Fisher Scientific, UK) was used to peel off the cortex from the remaining brain. An incision was made into the spine at the level of the hips and PBS was pushed into the spinal cavity using a 20 ml syringe (BD Plastipak, UK) fixed with a pipette tip (Fisherbrand, UK), ejecting the spinal cord from the rostral end of the spinal column.

Soleus/gastrocnemius muscles were removed together by dissection. Cortex, spinal cord and muscles were immediately snap frozen in liquid nitrogen and then stored at − 80 °C. Cortex and spinal cord from non-transgenic mice had been prepared using these methods from a previous cohort. Unfortunately non-transgenic snap frozen muscle tissue was unavailable^41^.

### RT-qPCR

RNA was extracted using an RNeasy® lipid tissue mini kit (74804, Qiagen, UK) as per the manufacturer’s instructions. Sample RNA concentrations were estimated using the Nanodrop spectrophotometer ND-1000 (Labtech International, UK). cDNA was extracted using a high capacity RNA-to-cDNA kit (4387406, Applied Biosystems, USA). Samples were placed in the PCR machine (G-storm, UK) for 1 h at 37 °C, followed by 5 min at 95 °C.

Primer pairs were mixed and diluted to 5 µM. Primers used were as follows: hTDP-43 (3′–5’ TGGGATGAACTTTGGTGCGT; 5′–3′ TTTGGCTCCCTCTGCATGTT), mouse (total) TDP-43 (3′-5’ TACCGGAATCCCGTGTCTCA; 5′–3′ GAGGAAGCATCTGTCTCATCCA), TBC1D1 (3′-5’ GTGAGGAAGAGGCGTTCAAG; 5′–3′ AGGTCTCGGTGGTAATCGTG), GAPDH (3′-5’ ATGGTGAAGGTCGGTGTGAA; 5′–3′ TGGCAACAATCTCCACTTTGC).

Samples were plated in duplicate in non-skirted, frosted, thin-wall 0.2 ml low profile 96 well plates (P3-0402, Geneflow, UK) and covered with optical wide area 8-cap strips (P3-0051, Geneflow, UK). Each well contained 1 µl cDNA, 1 µl primer mix, 3 µl water and 5 µl brilliant III Ultra-Fast SYBR® Green QPCR Master Mix (600882, Agilent Technologies, USA). GAPDH was used as the house-keeping gene. A non-template control was plated for each primer pair.

Samples were placed in the qPCR machine (Mx 3000P, Stratagene, USA) and run on the following protocol: 95 °C for 10 min, 45 cycles (95 °C for 30 s, 58 °C for 1 min, 72 °C for 1 min), 95 °C for 1 min, 55 °C for 30 s, 95 °C for 30 s.

Cycle threshold (Ct) values were extracted using MxPro Mx3000P software version 4.10 (Stratagene, USA). Further analysis was carried out using Microsoft Excel 2013 (Microsoft, USA) and GraphPad Prism 6 (GraphPad, USA).

Mean Ct values were calculated for each sample from the duplicates. Data were not used if duplicate Ct values differed by ≥ 1. The relative concentration was calculated using the equation: 1/2^(gene of interest Ct − GAPDH Ct)^. Values were presented as a percentage of the mean TDP-43^WT^ value for each sample, or as a percentage of the mean non-transgenic value wherever possible^41^.

### Western blotting

Samples (n = 3 male, 3 female/group, 10 months of age) were homogenised in RIPA buffer (150 mM sodium chloride, 50 mM Tris, 1% triton x-100, 0.5% SDS, 0.5% sodium deoxycholate, pH 8.0) and homogenised, followed by passage through a syringe and needle at least 3 times. Samples were then rolled at 4 °C for 30 min and left on ice for a further hour.

Samples were subjected to standard western blotting in 12% running gels and transferred to PVBDF membranes and probed with rabbit anti-TDP-43 primary antibody (10782-2-AP, Proteintech, UK, 1/1000) in 5% (w/v) milk/TBST. Mouse anti-β-actin primary antibody (ab6276, Abcam, UK, 1/1000) and anti-mouse HRP conjugate secondary antibody (W402B, Promega, UK, 1/5000) was used as a loading control and signal quantified using GeneTools software (Syngene, UK)^41^.

### Electrophysiology

Mice were placed under gaseous anaesthesia (1–2%, flow rate 1.0 L/min oxygen and nitrous oxide continuous inhalation through a nose cone). Body temperature was maintained using an electric heat pad (CWE, Ardmore, PA, USA) and a rectal temperature probe (Harvard Apparatus, Cambridge, UK) was used to supply feedback to the monitoring system (CWE, Ardmore, PA, USA). Compound muscle action potentials (CMAPs) were recorded using a Dantec Keypoint Focus EMG System. Supramaximal stimuli were applied subcutaneously at the sciatic notch, ring recording electrodes were placed circumferentially around the distal hindlimb muscles, as described by Arnold et al.^22^. EMG recordings were performed in gastrocnemius muscles using a concentric needle electrode (Ambu Neuroline, UK)^41^.

### Statistics

GraphPad Prism 6 was used for all statistical analyses (GraphPad, San Diego, CA, USA). Two-way ANOVA with Tukey’s post-test was used unless otherwise stated. All data are represented as a mean and standard deviation. Significance levels were set at 0.05, with two-tailed tests.

Power analysis was carried out using G*Power Version 3.1.9.2.^43^ using a Student’s *t* test, two-tailed, α = 0.05, β = 0.8 with equal N per group calculated for a range of putative effect sizes (10%, 15%, 20% difference from mean of the TDP-43^Q331K^ mice)^41^.

## Supplementary Information

Supplementary Information.

Supplementary Information 2.

Supplementary Information 2.

Supplementary Information 2.

## Data Availability

The datasets generated during and/or analysed during the current study are available from the corresponding author on reasonable request.
