# Supplementary material for: Extensive phenotypic characterisation of a human TDP-43Q331K transgenic mouse model of amyotrophic lateral sclerosis (ALS)
Source: Sci Rep. 2021 Aug 17;11:16659. doi: 10.1038/s41598-021-96122-z (PMC8370970; doi:10.1038/s41598-021-96122-z)
Supplement: Supplementary file 1 — Supplementary Information. [file 41598_2021_96122_MOESM1_ESM.docx]

**Supplementary Information**

**Extensive phenotypic characterisation of a human TDP-43^Q331K^ transgenic mouse model of amyotrophic lateral sclerosis (ALS)**

Jodie A. Watkins, James J. P. Alix, Pamela J. Shaw, Richard J. Mead

**Supplementary Movie 1 -TDP43^Q331K^ tremor.** This video shows the typical continuous resting tremor in a 6 month old TDP-43^Q331K^ female mouse. This phenotype develops from 10-14 weeks of age onwards.

**Supplementary Movie 2-TDP43^Q331K^ gait.** This video shows the obvious waddling or “swimming” gait in a 6 month old female mouse. This gait abnormality develops from approximately 27 weeks of age and can be quantified using gait analysis (see figure 2F and G)

**Supplementary Movie 3-TDP43^WT^ gait.** This video shows the normal gait observed in a TDP-43WT transgenic mouse at 6 months of age**.**


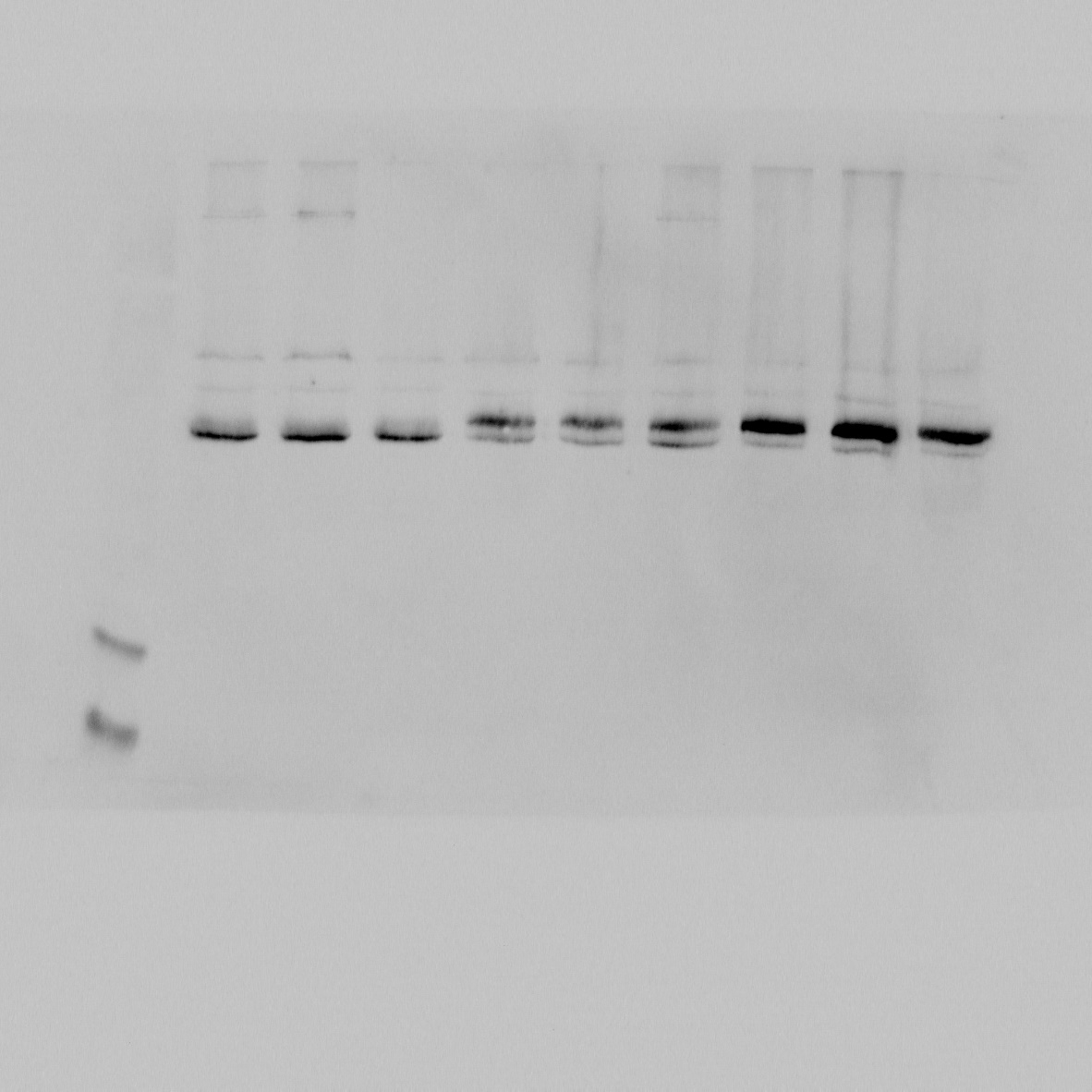


**Supplementary Figure 1**

Image showing the original captured image of the western blot in Figure 4d, TDP-43


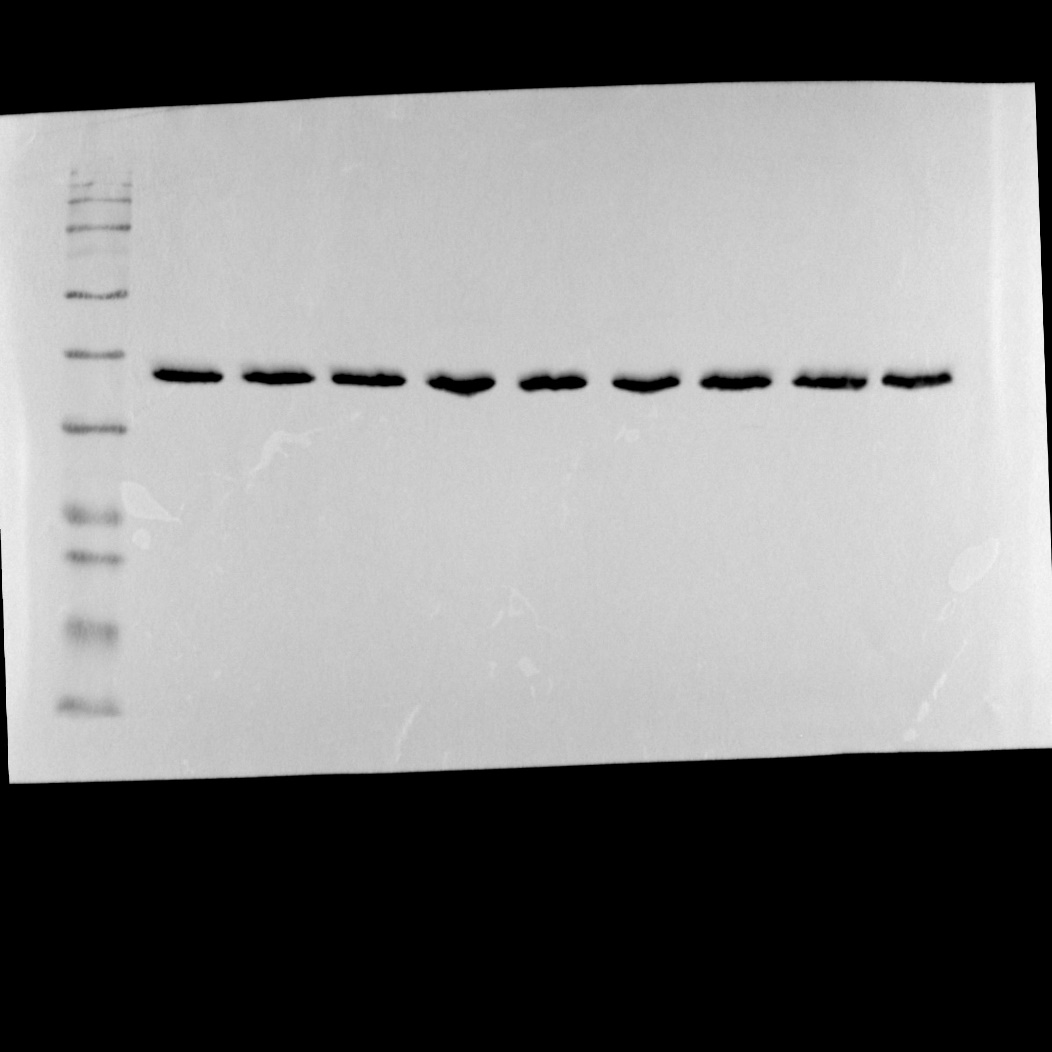


**Supplementary Figure 2**

Image showing the original captured image of the western blot in Figure 4d, beta-actin
